# Behavioral medicine in Teikyo University and Toho University

**DOI:** 10.1186/s13030-016-0057-5

**Published:** 2016-02-24

**Authors:** Takeaki Takeuchi, Masahiro Hashizume

**Affiliations:** Teikyo University Graduate School of Public Health & Department of Psychosomatic Medicine, Teikyo University Hospital, Tokyo, Japan; Department of Psychosomatic Medicine, Toho University School of Medicine, Tokyo, Japan; Department of Hygiene and Public Health, Teikyo University School of Medicine 2-11-1 Kaga, Itabashi, Tokyo 173-8605 Japan

**Keywords:** Behavioral medicine, Medical education, Psychosomatic medicine

## Abstract

Behavioral medicine has increased in importance to become a promising field in medical education. The Teikyo University Graduate School of Public Health and Toho University School of Medicine were evaluated in terms of their educational emphasis on behavioral medicine.

The Teikyo University Graduate School of Public Health has the following five core requirements, as in the global standards: behavioral medicine, biostatistics, epidemiology, occupational health, and health policy management. Behavioral medicine mainly encompasses psychology in normal populations, working as a gateway to the medical world among non-medical professionals who are interested in medicine. The Toho University School of Medicine aims to produce “good clinicians” who have a thorough knowledge, a deep sense of professional ethics, and a profound humanity to contribute to human welfare through medicine. In behavioral medicine here, systematic knowledge based on human behavior in medicine is taught from the first to sixth year.

Psychosomatic physicians could be among the most optimal professionals for behavioral medicine because of the similarities between psychosomatic medicine and behavioral medicine. The establishment of a Center of Behavioral Medicine is a potential solution to tackle forthcoming medical problems, such as increasing medical costs and an aging society. We must focus on the importance of behavior change as a way for preventive medicine to connect hospitals and communities in Japan.

## Background

Behavioral medicine has become a promising field of great importance. In medical education, the Education Commission for Foreign Medical Graduates (ECFMG) has decided that only foreign students who have graduated from medical schools certified internationally will be accepted to the medical board in the US after 2023 [1]. Therefore, the Japanese medical education committee has started to reform the medical education system in Japan. There is also an increasing trend in medical tourism and physician migration. In Japan, the committee of the Japan Society for Medical Education announced new standards in 2013— Basic Medical Education: Japanese Specifications World Federation for Medical Education Global Standards for Quality Improvement [2]. The Japan Accreditation Council for Medical Education will now establish the framework for accreditation of medical education in schools [3] and behavioral medicine will become a core curriculum in Japanese medical schools.

We selected two private schools: Teikyo University Graduate School of Public Health and Toho University School of Medicine. Teikyo University Graduate School of Public Health is the first public health graduate school that is an accredited independent entity and medical school. It contains global standards in five areas. Therefore it is one of the best public health schools having both a medical and an international perspective. Toho University School of Medicine established the first department of psychosomatic medicine in a Japanese private school in 1991. Therefore it takes the high road of psychosomatic medicine and behavioural medicine. We outline two cases of medical education in behavioral medicine at the Teikyo Graduate School of Public Health and Toho University School of Medicine that can help discuss the future of behavioral medicine and psychosomatic medicine in Japan.

### Behavioral medicine at the Teikyo Graduate School of Public Health

**Fig. 1:**
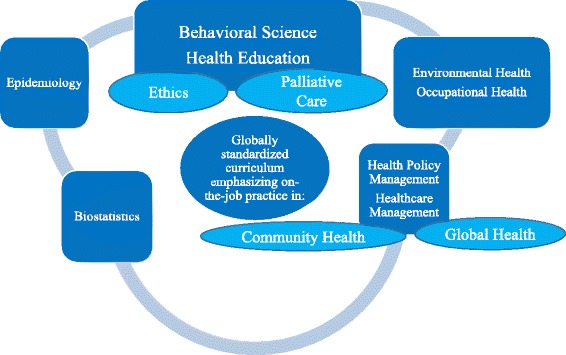
Teikyo Graduate School of Public Health

The Teikyo Graduate School of Public Health was established to provide a professional degree program in April 2011. It is the first public health graduate school that is an accredited independent entity and it contains global standards in five areas: behavioral sciences, biostatistics, environmental health sciences, epidemiology, and health policy and management (Fig. 1). Every January, five public health professors from Harvard University lecture and participate in short sessions called the Harvard Special Sessions. The aim of behavioral medicine at Teikyo is explained as follows. Problems due to miscommunication have been increasing in medicine. To tackle these problems, we must have adequate communication and good relationships with patients as well as pragmatic techniques based on evidence-based behavioral theory. Simultaneously, we need approaches to deal with both individual lifestyles and the patient’s environment. Therefore, our behavioral science classes have a pragmatic content, such as terminal care, behavioral science, health education, medical communication, and social epidemiology. Additionally, special lectures taught by professors from overseas are included to provide an international perspective. The syllabus is shown in Table 1 [4].

**Table 1:** Behavioral science (Teikyo University syllabus)

Subject	Behavioral science	Core	2 credits
Attainment target	Students who finish this course can		
	• explain fundamental theories of behavioral medicine.		
	• understand evidence-based knowledge and practice to apply behavioral medicine in the medical field.		
	• teach stress management to individuals and the wider population.		
Course summary	Students learn fundamental knowledge of behavior change and health promotion. They discuss the use of classical conditioning, operant conditioning, self-efficacy, and locus of control to evaluate and promote health. There is much evidence that cognitive behavioral medicine is effective for internal and psychiatric diseases, such as depression, social anxiety disorder, anorexia nervosa, lumber pain, hypertension, and diabetes. Students will gain state-of-the-art knowledge and techniques regarding behavioral medicine for stress reduction in Japanese society.		
Agenda	Contents		
	1	Introduction to behavioral science	
	2	Bio-psycho-social-spiritual model of behavioral science	
	3	Behavior change in medical practice	
	4	Relaxation response	
	5	Reflection of ourselves: understanding our mind	
	6	Body and mind relationships	
	7	Food and stress	
	8	Exercise and stress	
	9	Understanding of stress response	
	10	Cognitive behavioral therapy	
	11	Coping	
	12	Communication	
	13	Cognitive behavioral therapy in medicine	
	14	Group practice of behavioral medicine	
	15	Test and discussion of stress management	

Although our syllabus does not apply perfectly to the requirements of each university, it could be useful as a reference. Because classes are allocated under the public health curriculum, our syllabus is varied in its contents and includes school education and industrial psychology dealing with population health. The Teikyo Graduate School of Public Health is open to applicants from all fields and is not limited to those in medical school; therefore, it operates as a gateway to the medical world for non-medical professionals who are interested in medicine. Indeed, non-medical applicants have been increasing. Most classes cover basic psychology, rather than psychiatry, for patients; meanwhile, some classes teach psychiatry or pathological psychology at an advanced level. Two trained psychosomatic physicians, Professor Nakao and the author, teach these courses on behavioral medicine.

### Behavioral medicine at the Toho University School of Medicine

The Toho University School of Medicine aims to produce “good clinicians” who have a thorough knowledge, a deep sense of professional ethics, and a profound humanity to contribute to human welfare through medicine. A professional development education course operates from the first to the sixth year. This course focuses on encouraging good habits, not only in terms of learning medical knowledge, but also regarding independent problem solving and ethical behavior. In the first year, students learn health psychology as an introduction; in the fourth year, they learn psychosomatic medicine; and, in the sixth year, behavioral medicine (Table 2). In behavioral medicine, we try to teach systematic knowledge based on human behavior in medicine via psychosomatic physicians, psychiatrists, and pediatricians. We also change our class style from lectures to discussions. In the future, increased clinical training will increase pressure on the time spent on behavioral medicine-related classes and it will be important for us to manage our time well.

**Table 2:** Behavioral medicine (Toho University syllabus)

Course summary		
Behavioral medicine can be defined as the interdisciplinary field concerned with the development and integration of psychosocial, behavioral, and biomedical knowledge relevant to health and illness, and the application of this knowledge to prevention, etiology, diagnosis, treatment, and rehabilitation. Behavioral medicine is a field for studying human behavior and its background theory. It relates to psychology, sociology, and anthropology, and it focuses on communication and decision making. This class includes problem-based learning, small group learning, and role playing.		
Prerequisite knowledge and attitude		
1) Feeling and sensitivity as a human being		
2) Communication skills		
3) Understanding of medical professionalism		
4) Motivation to deal with patients systematically, not as a diseased being		
5) An understanding of stress based on biology, neurology, endocrinology, immunology, and psychiatry		
6) Completion of medical classes in somatic diseases		
Attainment target		
Students who complete this course can explain the following topics:		
• development process in life cycle		
• biological process of human behavior		
• psychological process of human behavior		
• psychological evaluation		
• major psychological therapies		
• dependency problems		
• sexual behavior and problems		
• meaning and disorders of violent behavior		
• suicidal conduct and its prevention		
• communication between patients and doctors		
Agenda	Contents	
	1	Introduction
	2	Anorexia nervosa
	3	Support for selected population (child)
	4	Support for selected population (adult)
	5	Sexual minority
	6	Support for selected population (different culture)
	7	Behavioral change (theory)
	8	Behavioral change (clinical case)
	9	Drug abuse
	10	Suicide prevention
	11	Sleep hygiene
	12	Abuse
	13	Professionalism
	14	Psycho-oncology
	15	Stress coping
	16	Support for selected population (aged)
	17	Terminal care: spiritual pain
	18	Terminal care: end of life

### Psychosomatic physicians and behavioral medicine

Psychosomatic physicians would be the optimum to teach behavioral medicine. The main reason would be the resemblance between psychosomatic medicine and behavioral medicine. According to the Japanese Society of Psychosomatic Medicine website, “Psychosomatic medicine is a study of the relationship between body and mind applying to medicine and it is broadly a systematic study dealing with psychological, social and environmental factors, not limited to psychosomatic diseases” [5]. Meanwhile, “Behavioral medicine is a systematic study dealing with factors such as social cultural, psychosocial, biomedical knowledge and techniques” [5]. For example, behavioral medicine applies to an adjunct treatment and establishing preventive healthy conduct, such as cognitive behavioral therapy (CBT), for psychosomatic diseases and mood disorders; it is used as an intervention for chronic diseases and life-related diseases. The field of behavioral medicine has been widening to solve the relationships between body and mind, clinical diagnosis and treatment, and public health [6].

While some people might narrowly define psychosomatic medicine as dealing with only psychosomatic diseases, generally, psychosomatic medicine and behavioral medicine are similar. Medical knowledge is therefore needed to teach at medical school or schools focusing on health, and psychosomatic physicians possess the potential to be behavioral medicine professionals. Assistance from psychologists, researchers of social psychology, and economic psychologists is needed. Psychosomatic physicians who know the “bio-psycho-social approach” teach the main flow of behavioral medicine.

### Future of behavioral medicine and psychosomatic medicine

When we think about the relationship between behavioral medicine and psychosomatic medicine, one problem is the unclear expertise of the psychosomatic physician. Is it psychiatry? Is it internal medicine? Or both? It is difficult to outline definitively the definition of psychosomatic medicine. In the 1980s, holistic medicine was increasing in importance, with some psychosomatic physicians playing a role in this. However, nowadays, general practitioners or family doctors play a major role in holistic medicine as well. Although psychosomatic medicine carries the banner of being “bio-psycho-social,” it is doubtful that this slogan is applicable to the Japanese medical situation. Where do psychosomatic physicians stand in the world when the special doctor license and medical education are globally standardized?

Japan is facing a rapidly aging society, medical inflation, and the “problem of 2025,” which have all been stressed for a long time [7]. To reduce medical costs, we should focus on the importance of behavior change as a means of preventive medicine; for example, consider the current situation in occupational medicine: here, overwork and suicide would be the main problems. A theory of early detection and early treatment is not sufficient; individual effort is needed to prevent illness and maintain a healthy condition. In other words, we must establish the framework to accomplish a healthy society by focusing on primary prevention. To manage these problems, the Ministry of Health, Labour and Welfare has some solutions: data health, collaborative health, and healthcare solution menus [8]. The Data Health Plan is an individual project within the scheme of specific health guidance measures aiming to clarify health issues via information and the establishment of effective healthcare projects that take into consideration the insurer’s function to address those issues. The PDCA (plan-do-check-act) cycle is an excellent method of managing this. Collabo health is a stratified health plan to prevent onset of disease by the company to which insured workers belong. The health care solution menu is an action to increase individual incentives to launch or maintain healthy activities based on individual lifestyle.

**Fig. 2:**
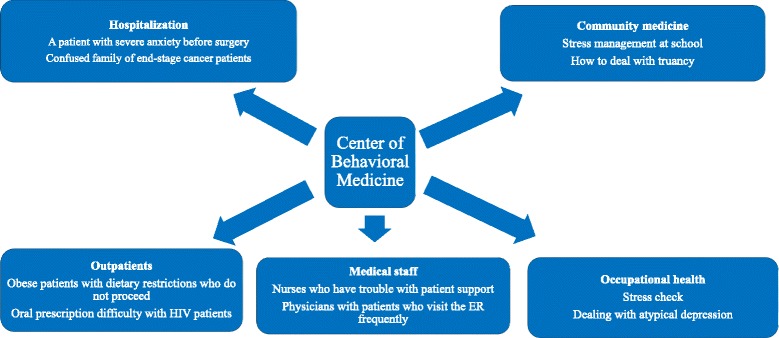
Proposed Center of Behavioral Medicine

However, these plans do not have any ability to extend beyond their existing functions. Therefore, the establishment of a Center of Behavioral Medicine would help put ideas into action. Behavioral medicine is a systemic study integrating the knowledge and techniques of social culture, social psychology, behavior, and biological medicine; therefore, it progresses through the axis of preventive medicine, health promotion, epidemiology, and treatment. An example of the proposed Center of Behavioral Medicine located at a university-affiliated hospital can be seen in Fig. 2. This center could support hospitalization, outpatients, community medicine, medical staff, and occupational medicine. There are some benefits in the establishment of the Center of Behavioral Medicine in many aspects, such as medical education, research, and clinical medicine. In medical education, students learn basic information about the theories of behavior, motivation, stress, lifelong development, and social and cultural factors to facilitate and maintain healthy behavior. In research, we can move toward establishing intervention studies for lifestyle diseases, occupational field studies, and prevention studies in the healthy population. In clinical medicine, the treatment outcome of lifestyle diseases improves and hospitals gain a positive reputation. The establishment of a Center of Behavioral Medicine as a medical bridge between the hospital and community will be a clear way for the success of behavioral and psychosomatic medicine. Both psychosomatic medicine and behavioural medicine are cutting-edge fields. They can carry out complementary functions in the future.

## Conclusions

Behavioral medicine has increased in importance to become a promising field in medical education. Two cases of behavioral medicine education exist at the Teikyo University School of Public Health and Toho University of School of Medicine. Psychosomatic physicians could be among the most optimal professionals for behavioral medicine. The establishment of a Center of Behavioral Medicine would be a new horizon for psychosomatic physicians to survive in the Japanese medical field.
